# Building a Broader Understanding of Enterotoxigenic *Escherichia coli*

**DOI:** 10.1093/ofid/ofaf376

**Published:** 2025-06-30

**Authors:** David A Sack, August L Bourgeois, Subhra Chakraborty

**Affiliations:** Department of International Health, Johns Hopkins Bloomberg School of Public Health, Baltimore, Maryland, USA; Department of International Health, Johns Hopkins Bloomberg School of Public Health, Baltimore, Maryland, USA; Department of International Health, Johns Hopkins Bloomberg School of Public Health, Baltimore, Maryland, USA

**Keywords:** Bangladesh, burden, disease, enterotoxigenic *E. coli*, epidemiology

The article by Chowdhury et al describing the epidemiologic and clinical features of enterotoxigenic *Escherichia coli* (ETEC) in Dhaka, Bangladesh, used the clinical and microbiological data from a large cholera vaccine trial in which patients with watery diarrhea sought clinical care at a facility [1]. Some of these patients did have cholera as reported earlier [2], but many did not. Fecal samples of all these patients were then screened for ETEC, and ETEC were found in some patients from whom *Vibrio cholerae* was isolated as well as those without detectable *V. cholerae*. This ETEC study benefited by its large population, which was followed for 4 years and included all ages and 2 cohorts: a “closed cohort” identified at the start of the trial (n = 174 731) and a “dynamic cohort” (n = 408 706). The latter accounted for changes in the population over time due to migration, births, and deaths. The rates in the closed cohort can inform the design of future vaccine trials for ETEC should these be planned, while the dynamic cohort better informs the overall disease in this urban area of Dhaka.

Several studies have attempted to estimate the burden of ETEC diarrhea in children [3], but there are limited population-based data regarding diarrhea rates that include all ages. Overall, the rate of ETEC was around 150 episodes per 100 000, but there was variability from year to year and by season. By having more cases during the pre- and postmonsoon times, seasonality was similar to that of cholera. An especially notable finding was the high rates in children aged <1 year; these infections were mostly associated with strains producing only heat labile toxin (LT). ETEC infections were also common in a cohort of Peruvian infants and children, but the strains in Peru were associated with either heat stabile toxin (ST) or ST and LT [4]. In the Dhaka study, the overall proportion of strains was similar among strains with LT only, LT and ST, and ST only. The authors also highlight the high number of older patients with severe dehydration. This high number was partly related to the larger number of people in this age strata in the population, but the proportion with moderate or severe dehydration was similar between those aged <5 and >14 years, being 187 and 146 per 100 000, respectively. Nevertheless, the study does highlight the importance of ETEC for all ages, not just those <5 years old.

ETEC diarrhea is often compared to cholera but, in general, is thought to be a less severe illness. However, this study found that 12% to 16% of the patients with ETEC without cholera had severe dehydration, which was especially common in those >45 years of age. Clearly, ETEC can cause severe diarrhea; in fact, ETEC were first discovered in patients with severe, cholera-like diarrhea from whom *V cholerae* could not be detected [5]. The spectrum of ETEC diarrhea, like cholera, can range from a relatively mild illness to one that causes life-threatening severe dehydration. Unlike cholera, which most often occurs during outbreaks and in specific geographic areas [6], ETEC is endemic in all low- and middle-income countries (LMICs) [7].

This study is also timely in providing data on ETEC's contribution to medically attended diarrhea in Bangladesh, which will help in the planning for future phase 2B and 3 studies for ETEC vaccines. Injectable protein vaccines are under development that include antigens for both toxins and the most common colonization factors [8]. In addition, an inactivated oral vaccine candidate that expresses these common colonization factors and the B subunit of LT (ETVAX) has completed phase 2 studies and is preparing for phase 3 trials in LMICs and travelers [9].

Because of the low attributable risk associated with LT-only strains observed in the GEMS studies [10, 11], some may downplay their importance as an important enteric pathogen. A previous birth cohort study from Bangladesh also reported higher rates of LT-only strains in control samples than diarrhea samples [12]. Clearly, LT-only strains can cause diarrhea as demonstrated by volunteer studies [13]. In addition, symptomatic as well as asymptomatic infections with LT-only ETEC are capable of inducing gut inflammation, which can facilitate enteropathy [14]. LT enterotoxin exposure may also serve to alter the structure and function of the intestinal epithelium, which may underlie the acute and long-term negative health outcomes associated with ETEC infection among young children in LMICs [15]. Moreover, LT has the potential to modulate receptor expression in the intestine, as well as multiple other aspects of gut physiology that render individuals who are infected more susceptible to other enteric pathogens [16–18].

The article from Dhaka had some limitations; the most striking was the lack of data on the colonization factors expressed by the ETEC strains. Current ETEC vaccine candidates are based on stimulating immunity to these colonization factors and their toxins. There are at least 25 immunologically heterogeneous adhesins produced by ETEC strains to attach bacteria to host receptors to colonize small intestines, but of these, 7 (CFA/1 and CS1-6) are associated with most moderate and severe cases [19]; thus, candidate vaccines target these colonization factors. A second caution is related to the study being carried out in 1 large urban area in Bangladesh and the data now being 10 years old. Vaccine studies will need to be conducted in different locations and in different settings to ensure broad protection.

Chowdhury and colleagues close their article emphasizing the need for continued efforts to develop better prevention and control strategies for ETEC in endemic areas. As with cholera, these strategies must include improved water and sanitation, but as with cholera, a vaccine also plays a key role. The overlapping epidemiology of ETEC and cholera described in their article makes a strong public health case for the development of combination vaccine approaches that could target ETEC and cholera. In a similar vein, the recent World Health Organization vaccine value profile for ETEC suggests that the value proposition for ETEC vaccine development and uptake would be significantly increased if it could be used in a combination vaccine with other enteric pathogens, such as cholera and/or *Shigella*. Combination vaccine options would not only reduce the risk of acute illness associated with these pathogens but should also combat other negative health outcomes more effectively, such as stunting and antimicrobial resistance development [20, 21]. The polymicrobial nature of both these public health concerns might be better addressed with a combination approach. Hopefully, the data presented by Chowdhury and colleagues in this article will encourage vaccine developers to more seriously consider the combination vaccine approach.

## Notes

***Financial support.*** This work was supported by the National Institute of Allergy and Infectious Disease to D. A. S. (grant 5R01AI123422) and to S.C. (grants RO1AI153399 and R56AI168316).
